# PEGylated arginine deiminase can modulate tumor immune microenvironment by affecting immune checkpoint expression, decreasing regulatory T cell accumulation and inducing tumor T cell infiltration

**DOI:** 10.18632/oncotarget.19564

**Published:** 2017-07-26

**Authors:** Elena Brin, Katherine Wu, Hsin-Tze Lu, Yudou He, Zhaoming Dai, Wei He

**Affiliations:** ^1^ Polaris Pharmaceuticals, San Diego, CA, USA; ^2^ DesigneRx Pharmaceuticals, Shanghai, China

**Keywords:** arginine deiminase, TILs, Treg, immunomodulation, PD-L

## Abstract

PEGylated arginine deiminase (ADI-PEG 20) is being investigated in clinical studies in arginine auxotrophic cancers and is well-tolerated. The anti-tumor properties of ADI-PEG 20 have been extensively investigated - ADI-PEG 20 inhibits the growth of auxotrophic cancers *in vitro* and *in vivo* - however, its impact on immune cells is largely unknown. Here we report the potential impact of ADI-PEG 20 on the tumor immune microenvironment. ADI-PEG 20 induced immunosuppressive programmed death-ligand 1 expression on some cancer cells *in vitro*, but the magnitude of the increase was cell line dependent and in most relatively small. Using healthy donor human peripheral blood mononuclear cells (PBMCs) we discovered that when present during initiation of T cell activation (but not later on) ADI-PEG 20 can inhibit their differentiation after early activation stage manifested by the expression of CD69 marker.

*In vivo*, ADI-PEG 20 induced tumor T-cell infiltration in a poorly immunogenic syngeneic mouse melanoma B16-F10 model and reduced its growth as a single agent or when combined with anti-PD-1 mAb. It was also effective by itself or in combination with anti-PD-L1 mAb in CT26 colon carcinoma syngeneic model.

## INTRODUCTION

Unlike normal tissues many tumors cannot synthetize arginine from citrulline due to deficiency of the key urea cycle enzyme, argininosuccinate synthetase 1 (ASS1) and this arginine auxotrophy phenomenon has been explored with anti-cancer treatments that deplete arginine [1–7]. PEGylated arginine deiminase (ADI-PEG 20) depletes the external supply of arginine by converting it to citrulline and ammonia. ADI-PEG 20 is being investigated in the clinic for the treatment of patients with tumor deficiency in ASS1. ASS1 is required for the conversion of citrulline to arginine [8]. ADI-PEG 20 has been demonstrated to be well-tolerated and has shown promise in clinical studies [1–7].

Arginine is used in a number of metabolic pathways, including those involved in modulating immune responses. It is a substrate for arginase and iNOS, enzymes that play important roles in immune cell regulation [9–15]. T cells need arginine for proliferation, T cell receptor (TCR) expression, and development of memory [9–12].

While arginine deprivation has been shown to impede proliferation and cell cycle progression of activated T cells *in vitro* [16–20] this effect can be reversed by addition of citrulline [16–18]. Similarly, prolonged loss of CD3 and T cell inhibition was induced *in vitro* by macrophages expressing arginase I (converts arginine to ornithine) and not those producing NOS2 (converts arginine to citrulline and NO) [21]. Lack of arginine induces increased transcription and stability of RNAs encoding a multi–amino acid transport system including CAT-1 which increases transport of cationic amino acids into the cell [22–25]. Under low arginine conditions T cells increase citrulline uptake and upregulate expression of ASS1 which converts citrulline into arginine [16, 17, 26]. Therefore, while conversion of arginine into ornithine by arginase can lead to immunosuppression in the tumor microenvironment [17] conversion of arginine into citrulline may have a different outcome.

The anti-tumor properties of ADI-PEG 20 have been extensively investigated [3–7, 27–29], however, its impact on immune cells is largely unknown. Therefore, we have investigated potential implications of ADI-PEG 20 treatment on tumor immune surveillance. We analyzed the effect of ADI-PEG 20 on T cell subsets in healthy donor peripheral blood mononuclear cells (PBMCs) under resting and activation conditions. ADI-PEG 20 did not affect resting T cells. Under stimulation conditions while allowing CD69 upregulation ADI-PEG 20 inhibited further T cell activation including expression of checkpoint inhibitor molecules and accumulation of cells with regulatory T cells (Treg) markers (CD3+CD4+CD25+FoxP3+CTLA4+). When ADI-PEG 20 treatment was initiated more than a day after stimulation it did not affect T cells.

Immune checkpoint inhibitor programmed death ligand 1 (PD-L1) can dampen immune responses and its expression on tumor cells can be affected by various factors. We found that ADI-PEG 20 upregulates PD-L1 in a number of cancer cell lines *in vitro*, but the magnitude of the increase is cell line dependent and in the majority of the studied cell lines it was rather small.

We investigated ADI-PEG 20 efficacy in syngeneic models by itself and in combination with anti-mPD-1 and mPD-L1 antibodies. In a poorly immunogenic syngeneic B16-F10 melanoma mouse model ADI-PEG 20 treated animals had a large T cell infiltrate while, as expected, very few T cells were found in tumors from non-treated controls. ADI-PEG 20 reduced tumor growth in both B16-F10 and CT26 models and trended to be additive with anti-mPD-1 and mPD-L1 antibodies.

## RESULTS

### Effect of ADI-PEG 20 on PD-L1 expression in ASS1-low cancer cell lines

We have selected 16 ASS1-low cancer cell lines as well as a control ASS1-high cell line (N87) to investigate the effect of ADI-PEG 20 treatment on the expression of the well-characterized immune checkpoint inhibitor PD-L1. ASS1 levels were determined by Western Blotting and qPCR analyses (data not shown).

Cells were treated with increasing concentrations of ADI-PEG 20 or with 200 ng/mL IFNγ for 24 h, 48 h or 72 h. At each time point treated and non-treated cells were collected and surface stained with anti-PD-L1 mAb or isotype control antibody and co-stained with Live/Dead fixable stain as described in Materials and Methods. To correct for non-specific binding we subtracted values obtained with isotype control antibody from those acquired after staining with anti-PD-L1 antibody.

**Figure 1:**
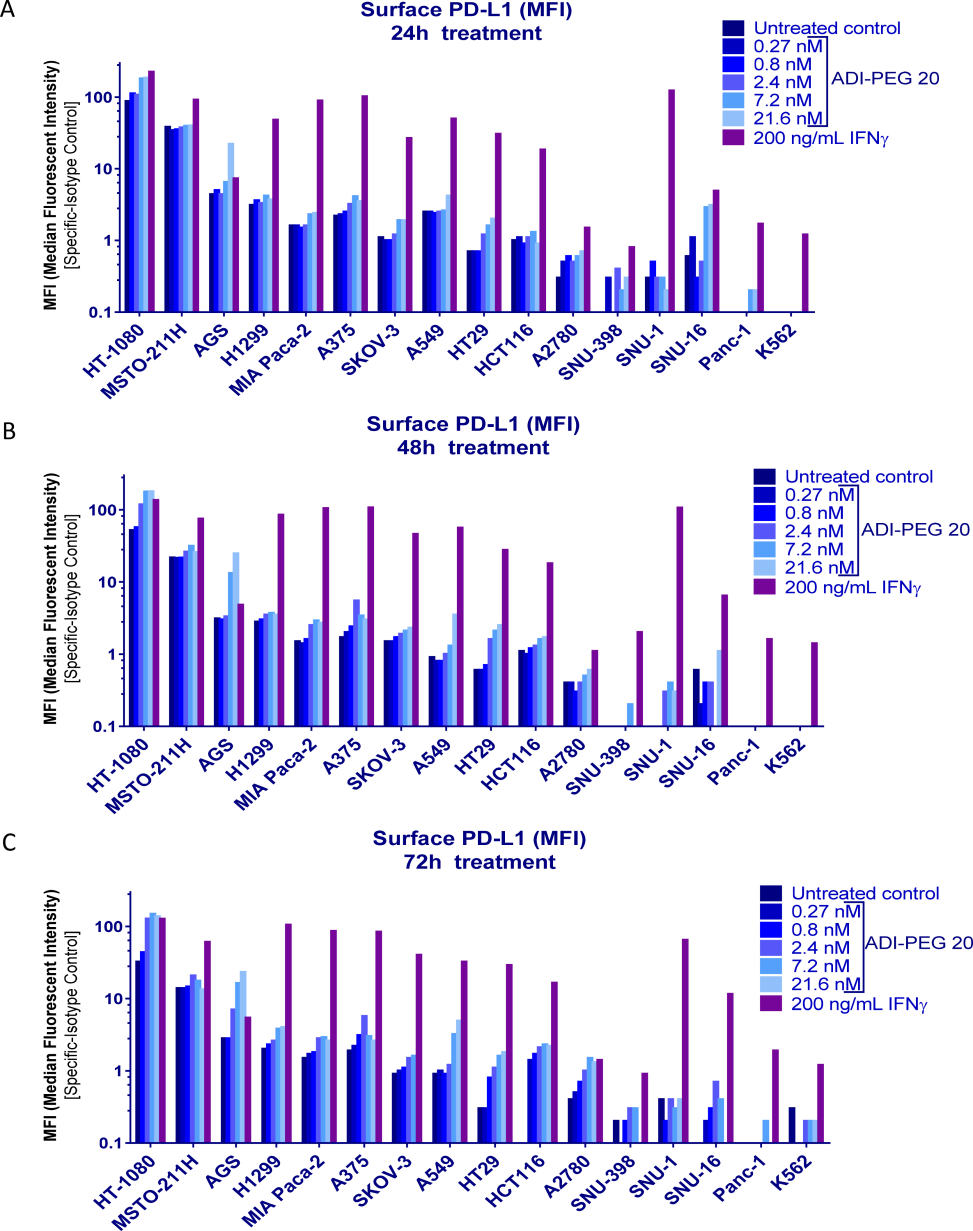
Effect of ADI-PEG 20 on PD-L1 cell surface levels in a cancer cell panel Cancer cells were treated with serially diluted ADI-PEG 20 or IFNγ control and their surface PD-L1 was assessed by flow cytometry at 24 h (**A**), 48 h (**B**) and 72 h (**C**) after the start of the treatment.

A summary of the results is shown in Figure 1. In the majority of the tested cells lines 200 ng/mL IFNγ had greater effect on PD-L1 upregulation than ADI-PEG 20. Only in one cell line, AGS, treatment with high concentrations of ADI-PEG 20 upregulated PD-L1 more than observed for treatment with 200 ng/mL IFNγ. ADI-PEG 20 and 200 ng/mL IFNγ had similar effects in HT-1080 cells further upregulating an already high PD-L1 level, and in PD-L1-low A2780 cells both agents induced only minor increase in PD-L1 (ADI-PEG 20 effect seen mainly at 72 h). In H1299 and Mia Paca-2 ADI-PEG 20 upregulated PD-L1 only slightly and in A375, SKOV-3, HT29, and HCT116 ADI-PEG 20 increased surface PD-L1 several fold while 200 ng/mL IFNγ increased it 20 fold or higher (based on median fluorescent intensity (MFI) values). ADI-PEG 20 had little to no effect on PD-L1 levels in SNU-398, SNU-16, Panc-1, K562 and SNU-1 cell lines. Treatment with 200 ng/mL IFNγ greatly upregulated PD-L1 levels in SNU-1 and only modestly in SNU-398, SNU-16, Panc-1 and K562. As expected, in the ASS1-high (negative control) N87 cell line ADI-PEG 20 had no effect whereas 200 ng/mL IFNγ prominently upregulated PD-L1 (data not shown).

**Figure 2:**
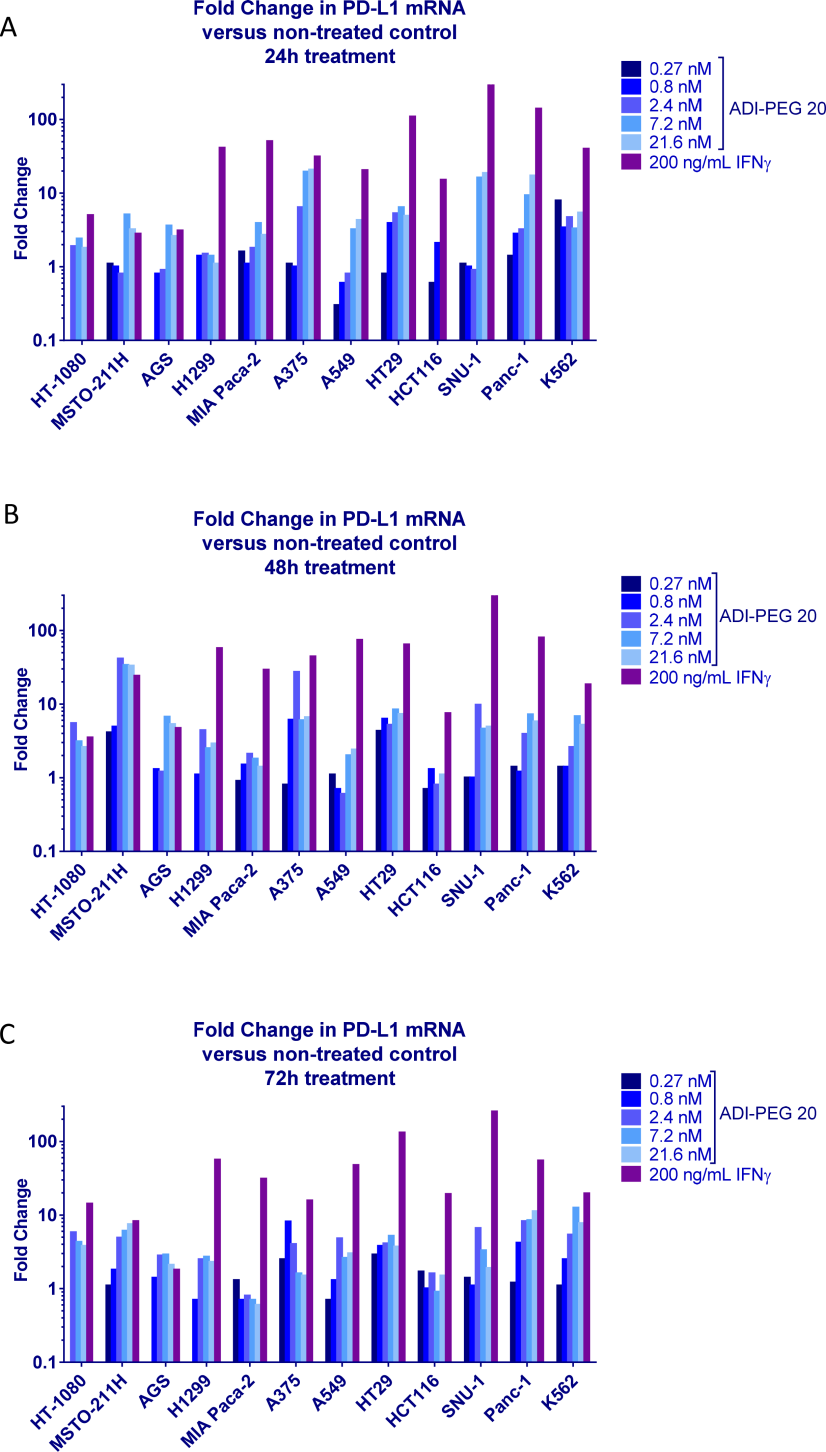
Effect of ADI-PEG 20 on PD-L1 expression in a cancer cell panel Cancer cells were treated with serially diluted ADI-PEG 20 or IFNγ control and PD-L1 mRNA levels were measured by RT-qPCR at 24 h (**A**), 48 h (**B**) and 72 h (**C**) after the start of the treatment. β-actin mRNA was used for normalization.

PD-L1 encoding CD274 gene expression data by RT-qPCR generally paralleled PD-L1 cell surface level findings by flow cytometry; results are summarized in Figure 2.

These data suggest PD-L1 upregulation was at least partially driven by gene expression.

### ADI-PEG 20 treatment does not affect resting PBMCs but when present during initial stimulation inhibits T cell activation past CD69 upregulation stage

PBMCs were rested overnight and treated for 24 h, 48 h or 72 h with 0–20 nM ADI-PEG 20 in the presence or absence of anti-CD3/CD28 beads or Phytohaemagglutinin (PHA). To control for any potential effects of polyethylene glycol (PEG 20) and verify that the observed treatment outcomes with ADI-PEG 20 were indeed mediated by ADI activity we have evaluated the effect of 20 nM of an inactive ADI mutant (with C397A substitution) PEGylated with 20K PEG.

**Figure 3:**
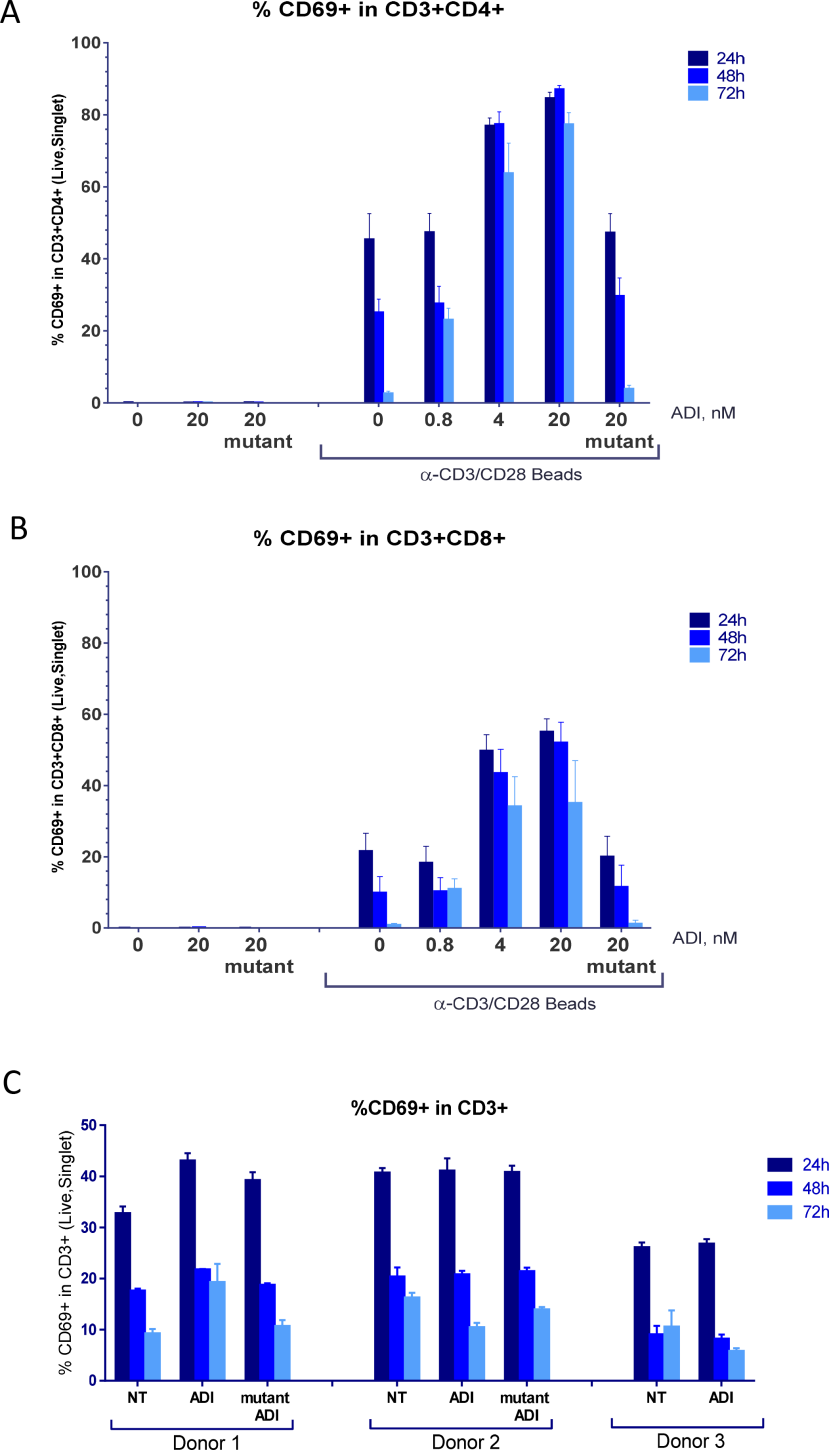
ADI-PEG 20 treatment during PBMC stimulation and not under resting conditions lead to a sustained increase in CD69+ T cells PBMCs were stimulated with anti-CD3/CD28 Dynabeads in the presence or absence of ADI-PEG 20 or mutant ADI-PEG 20. Percentages of CD69+ cells among CD4+ T cells (**A**) and CD8+ T cells (**B**) were determined by flow cytometry at 24 h, 48 h & 72 h. PBMCs containing CD69+ T cells in the absence of stimulation were not affected by 20 nM ADI-PEG 20 or mutant ADI-PEG 20 (**C**). NT – non-treated.

**Figure 4:**
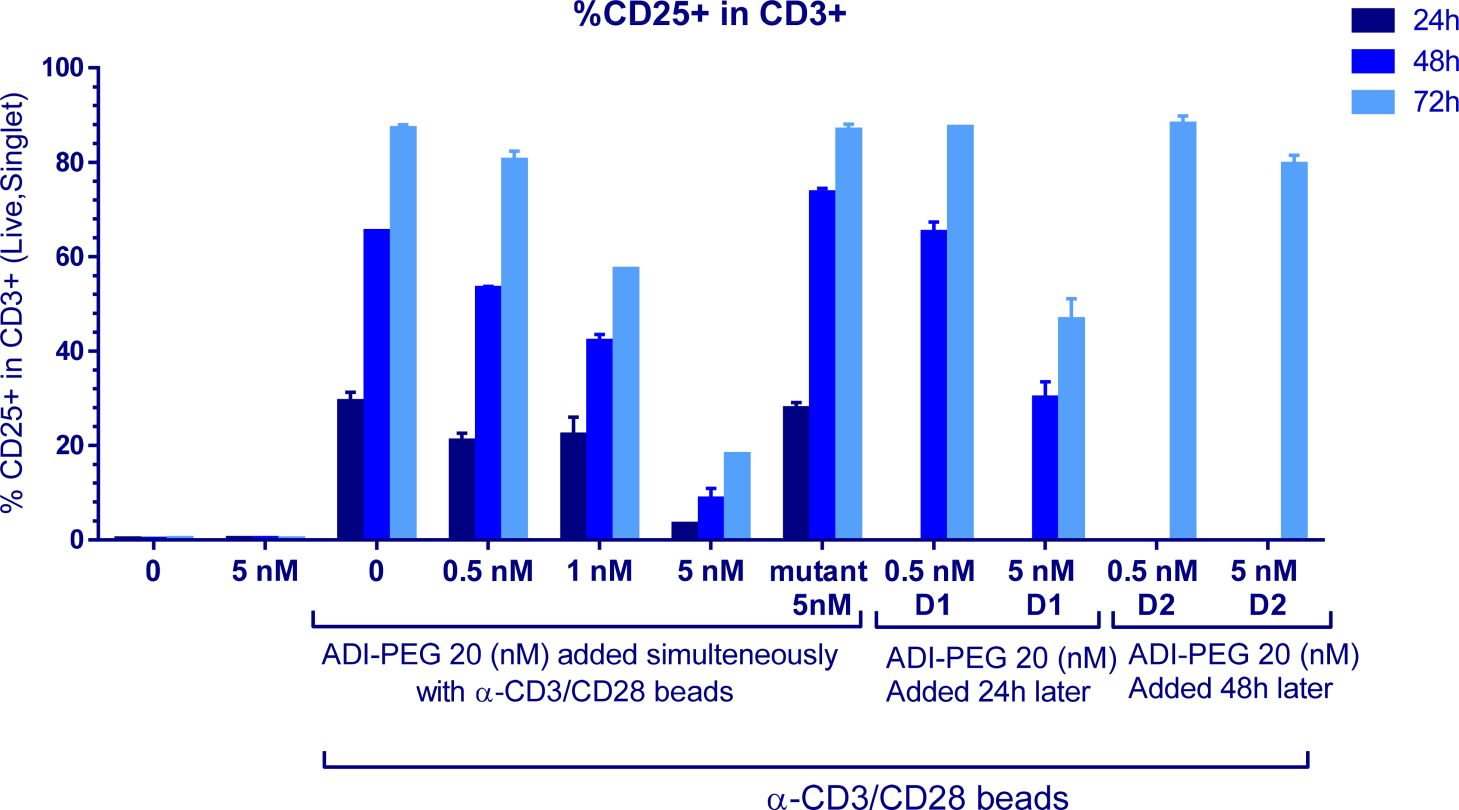
CD25+ T cell induction was affected by high (and not low) concentrations of ADI-PEG 20 when added during PBMC stimulation and not under resting conditions or when added 48 h after the addition of anti-CD3/CD28 Dynabeads

**Figure 5:**
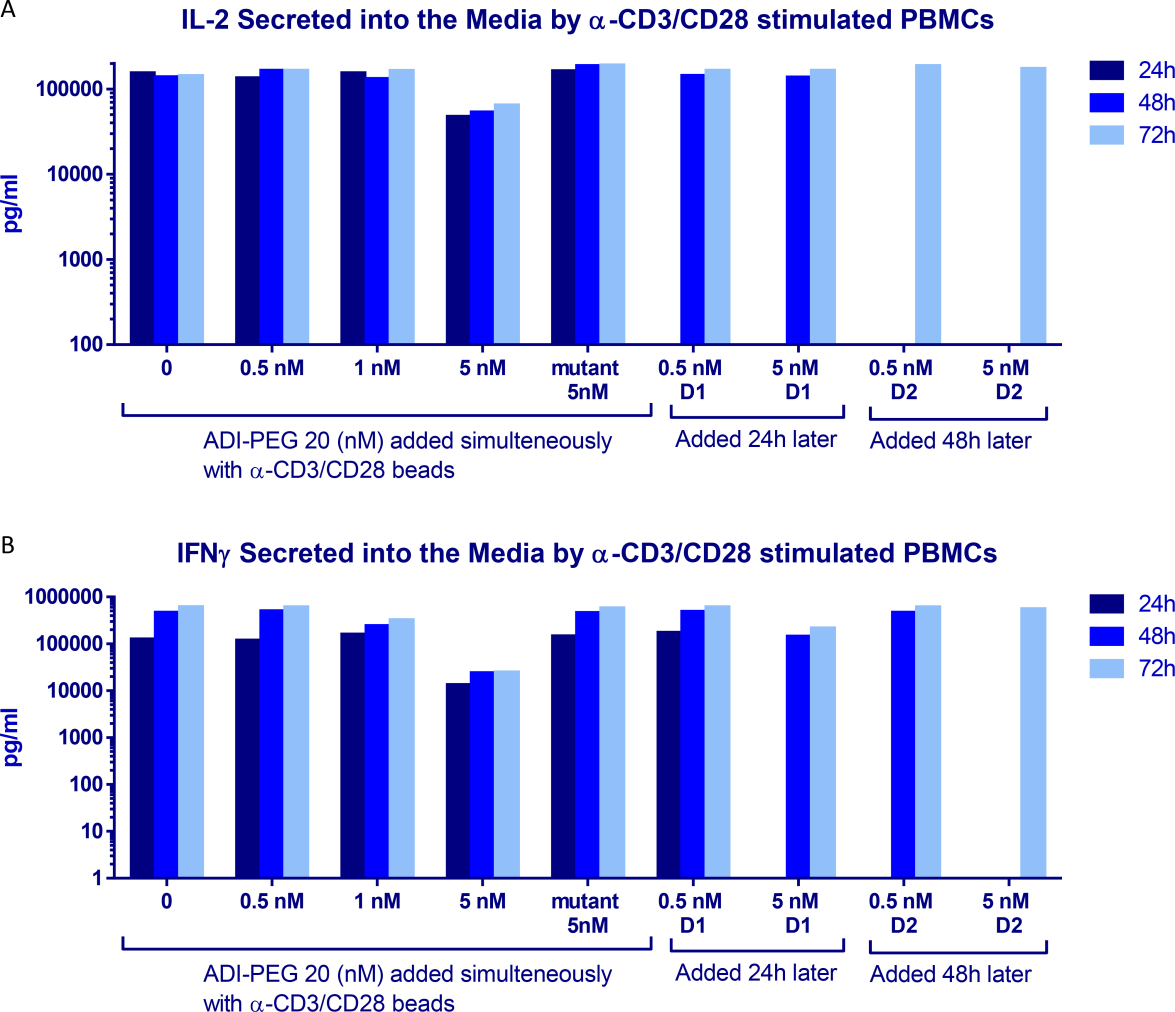
IL-2 (**A**) and IFNγ (**B**) secretion by PBMCs after stimulation with anti-CD3/CD28 Dynabeads in the presence or absence of ADI-PEG 20 or mutant ADI-PEG 20. ADI-PEG 20 was added simultaneously with the beads or 24 h or 48 h later. IL2 and IFNγ in the media were measured by ELISA at 24 h, 48 h and 72 h after initiation of the stimulation.

**Figure 6:**
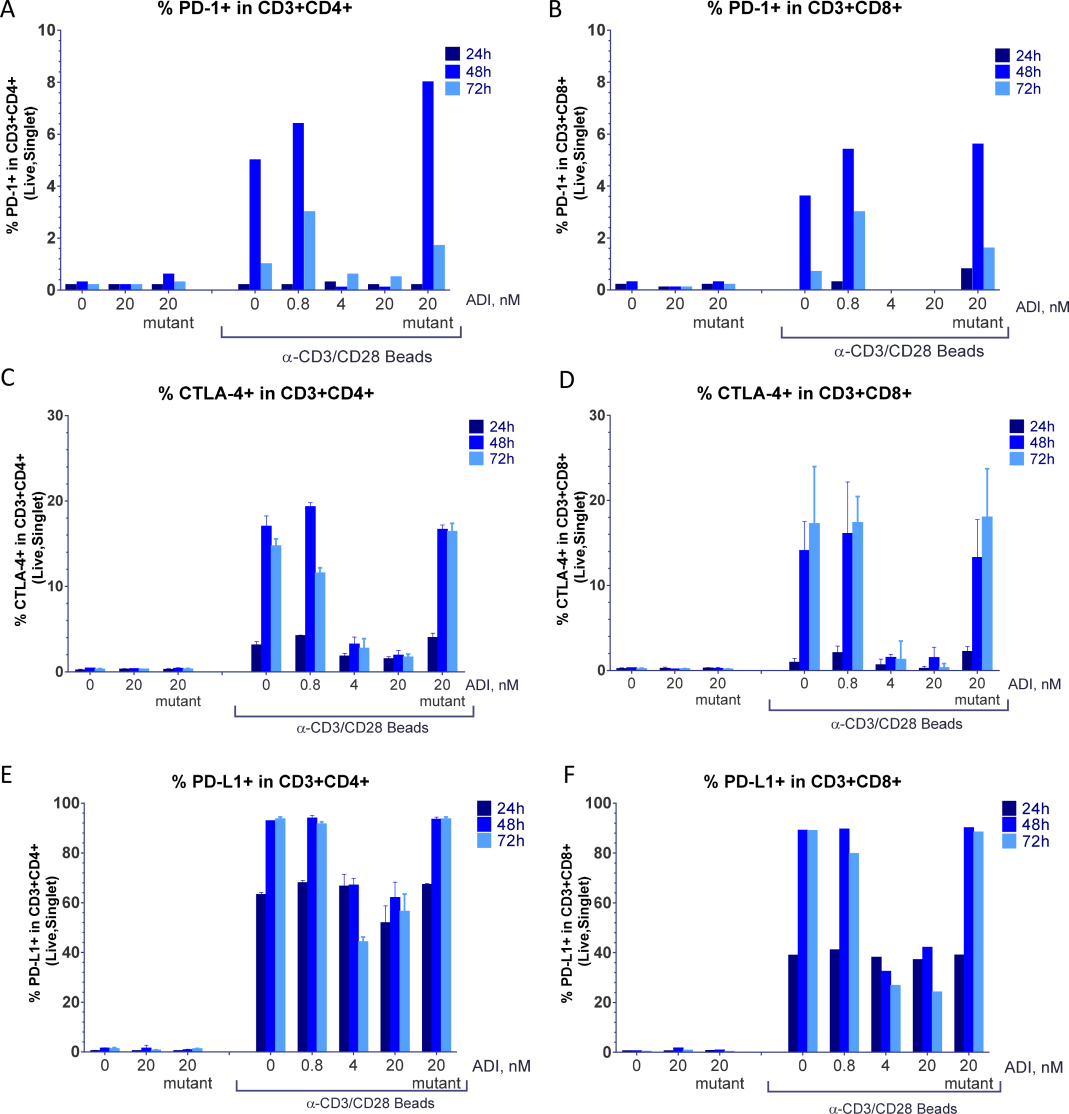
ADI-PEG 20 blocks anti-CD3/CD28 induced upregulation of PD-1, CTLA-4 and PD-L1 on T cells PBMCs were stimulated with anti-CD3/CD28 Dynabeads in the presence or absence of ADI-PEG 20 or mutant ADI-PEG 20. Percentages of PD-1+ (**A**–**B**), CTLA-4+ (**C**–**D**) and PD-L1+ (**E**–**F**) cells in CD4+ T cells (A, C, E) and CD8+ T cells (B, D, F) were determined by flow cytometry at 24 h, 48 h & 72 h.

**Figure 7:**
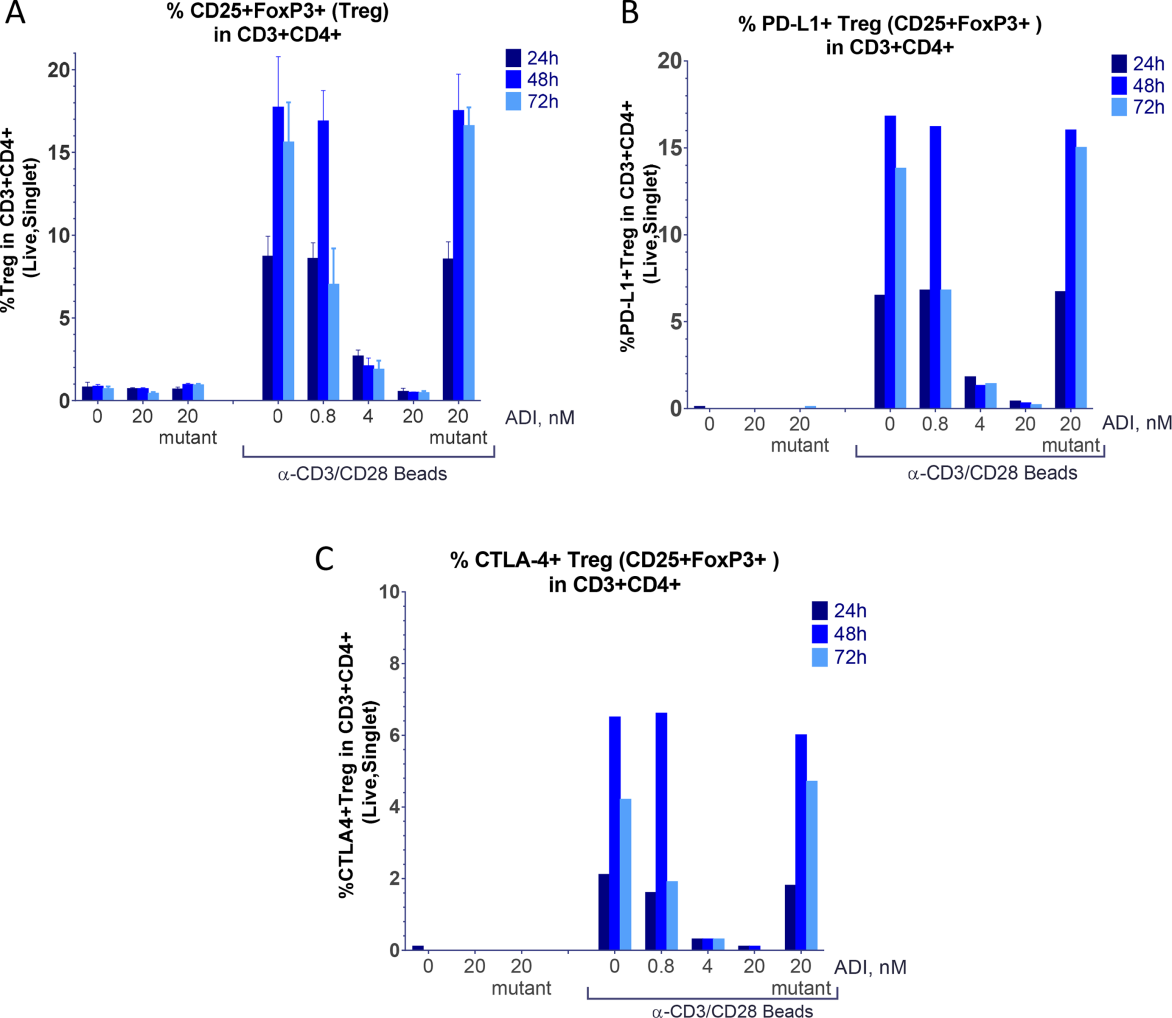
ADI-PEG 20 inhibits accumulation or T cells with Treg markers PBMCs were stimulated with anti-CD3/CD28 Dynabeads in the presence or absence of ADI-PEG 20 or mutant ADI-PEG 20. Percentages of Treg cells among CD4+ T cells were determined by flow cytometry at 24 h, 48 h & 72 h. Treg cells were determined by CD3+CD4+CD25+FoxP3+ (**A**), CD3+CD4+CD25+FoxP3+PD-L1+ (**B**) or CD3+CD4+CD25+FoxP3+CTLA-4+ (**C**) markers.

After incubation was complete cells were collected, stained for expression of surface markers and analyzed by immune cell phenotyping using flow cytometry. Six independent experiments were conducted with PBMCs from five different donors and the obtained results were consistent between experimental repeats. Representative data are shown in Figures 3-7.

ADI-PEG 20 treatment of PBMCs under resting conditions had no effect on the analyzed T cell subsets (Figures 3-7).

When ADI-PEG 20 treatment was initiated simultaneously with anti-CD3/CD28 bead addition to PBMC cultures CD4+ and CD8+ T cells expressing early activation marker CD69+ persisted throughout the treatment while in the absence of ADI-PEG 20 or in the presence of mutant ADI-PEG 20 they have subsided (as expected) as shown in Figure 3A and 3B. This effect was more apparent at the high concentrations of ADI-PEG 20 which very quickly convert all media arginine into citrulline. The 0.8 nM ADI-PEG 20 dose did not have a noticeable effect at the 24 h and 48 h time points and blocked CD69 downregulation at 72 h, which reflects slower depletion of arginine at this concentration. Kinetics of arginine conversion into citrulline in the media by a range of ADI-PEG 20 concentrations are shown in Supplementary Figure 1.

PBMCs from three of the five donors had relatively high percentage of CD69+ T cells in the absence of activation. CD69 expression has decreased overtime as in freshly stimulated cultures and in this case ADI-PEG 20 did not affect the downregulation as shown in Figure 3C.

Next, we analyzed percentage of T cells expressing another activation marker CD25 that is upregulated later than CD69 and remains high for a few days. When added simultaneously with anti-CD3/CD28 beads high (but not low) concentrations of ADI-PEG 20 reduced CD25+ T cells. When 5 nM ADI-PEG 20 was added 24 h after the initiation of stimulation reduction in CD25+ T cells was very modest and addition of 5 nM ADI-PEG 20 48 h after activation had no effect on CD25 expression as shown in Figure 4.

Consistent with these findings IL-2 and IFNγ secretion into media by anti-CD3/CD28 stimulated PBMCs was decreased when 5 nM ADI-PEG was present from the beginning of T cell activation, but not when added 24 h or 48 h after bead addition or when used at lower concentrations (Figure 5). 5 nM ADI-PEG 20 rapidly depletes all arginine from the media while 0.5–1 nM concentrations convert arginine to citrulline more gradually (Supplementary Figure 1) leaving some (albeit low) arginine available during early activation events (at the beginning of the treatment).

Induction of PD-1 and CTLA-4 on CD4+ and CD8+ T cells starting at 48 h following stimulation was prevented by the treatment with high concentrations of ADI-PEG 20 (Figure 6A–6D). In the absence of stimulation or when added 48 h later than anti-CD3/CD28 beads ADI-PEG 20 had no effect on PD-1 and CTLA-4 expression (data not shown).

PD-L1 surface levels on T cells were greatly increased following anti-CD3/CD28 bead (or PHA) stimulation with surface PD-L1 detectable on almost all T cells by 48 h. ADI-PEG 20 had little to no effect on PD-L1 levels at 24 h timepoint but when present at high concentrations (4 or 20 nM) it blocked further upregulation of PD-L1 in CD4+ and CD8+ T cells (Figure 6E–6F).

Non-functional mutant ADI-PEG 20 did not affect T cells under either resting or stimulating conditions.

### ADI-PEG 20 inhibited induction of cells with regulatory T cell markers

Regulatory T cells are known to have CD3+CD4+CD25+FoxP3+CTLA-4+ phenotype. CD4+ T cells differentiation into CD3+CD4+CD25+FoxP3+ cells was inhibited by ADI-PEG 20 in a concentration dependent manner (Figure 7). ADI-PEG 20 at the 4 nM and 20 nM doses almost completely blocked induction of CD3+CD4+CD25+FoxP3+ T cells including those that co-expressed PD-L1 and CTLA-4 markers (Figure 7).

In preliminary experiments we have also included CD127 marker for identification of regulatory T cells and the results were similar whether or not gating on CD127- cells was included (almost all CD3+CD4+CD25+FoxP3+ were CD127-, data not shown).

Similar to the T cell activation data (CD69 levels) the 0.8 nM ADI-PEG 20 dose was not effective after 24 h or 48 h of treatment but reduced cells with regulatory T cell markers at the 72 h time point.

PBMC viability was not reduced by ADI-PEG 20 as shown in Supplementary Figure 2.

PBMC proliferation was not affected under non-stimulated conditions while in the presence of anti-CD3/CD28 beads ADI-PEG 20 inhibited proliferation when present at 1–5 nM concentration but not at 0.5 nM (Supplementary Figure 3).

### ADI-PEG 20 induced T cell infiltration in B16 melanoma model

**Figure 8:**
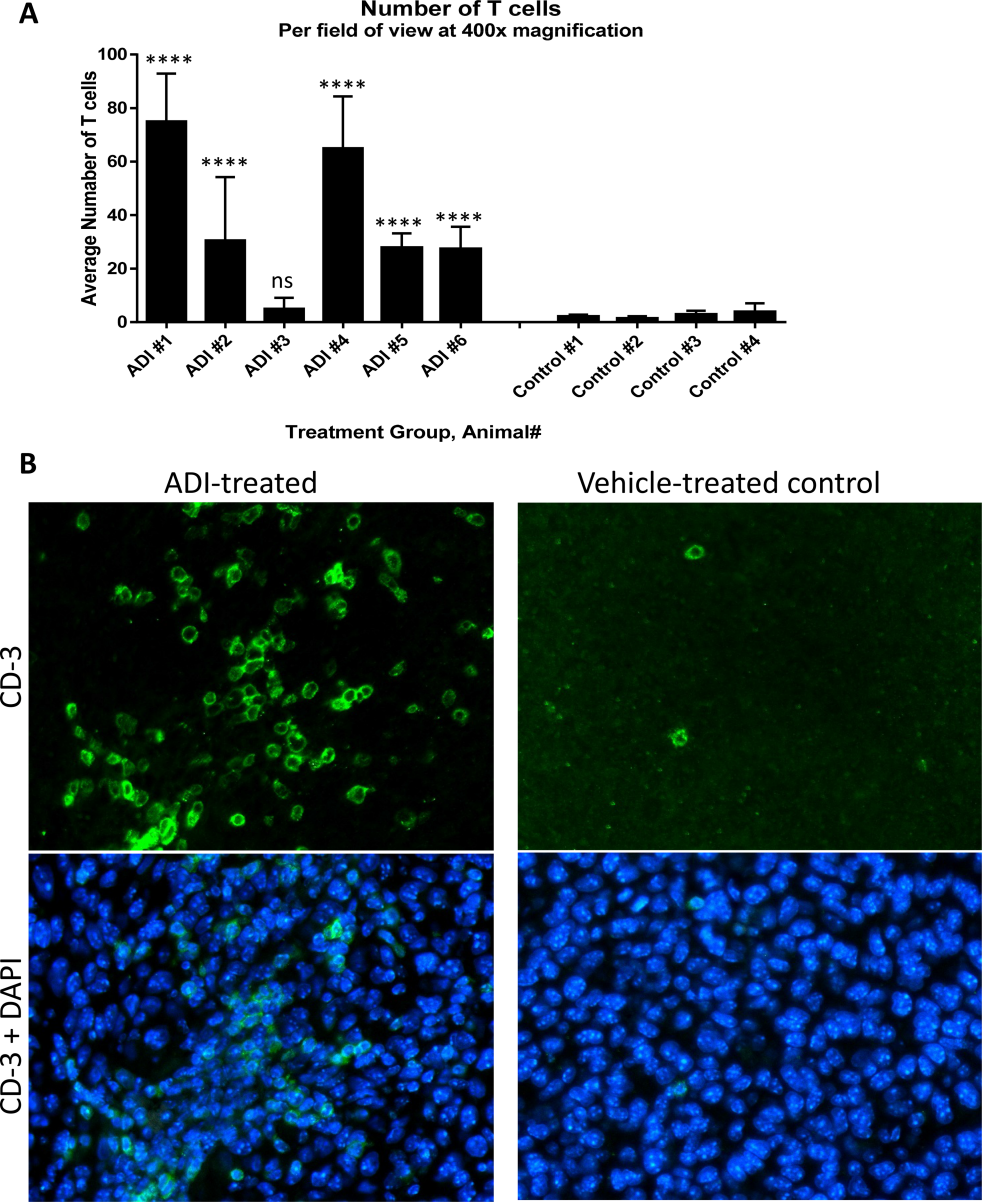
ADI-PEG 20 induces T cell infiltration into B16-F10 tumors B16-F10 cells were implanted into C57BL/6 mice and the mice were treated IM with 12 mg/kg of ADI-PEG 20 or vehicle (PBS) control on d1 and d7 post tumor implantation. On day 14 tumors were removed and sectioned. Sections were stained by IHC with anti-CD3 mAb and counterstained with DAPI. Number of T cells per field at 400× was counted in four sections of each tumor and averaged (**A**), representative images taken at 400× magnification (**B**). One-way Anova was used for statistical analysis, ns – non significant, ****p < 0.0001.

C57BL/6 mice were implanted with B16-F10 melanoma cells and treated, as described in Materials and Methods, by intramuscular injections with either PBS (control group) or 12 mg/kg of ADI-PEG 20 (experimental group); treatments were administered on day 1 and day 7 after tumor implantation. On day 14 after tumor implantation animals were euthanized and their tumors were removed and paraffin-embedded for IHC analysis. Tumor sections were stained with anti-CD3 mAb to determine whether ADI-PEG 20 treatment promoted T cell infiltration into the tumors. As expected, the PBS-treated animals had very few T cells present in their tumors. Conversely, five out of six ADI-PEG 20 treated animals had a large presence of T cells in their tumors which was statistically significant (*p* < 0.0001) when comparing to the average number of T cells present in the tumors of control animals using one-way Anova with Dunnett's test. Average number of TILS per tumor section is shown in Figure 8A and representative images in Figure 8B. Both CD4+ and CD8+ T cells were present among the infiltrating T cells (data not shown).

### ADI-PEG 20 inhibits tumor growth in syngeneic models

**Figure 9:**
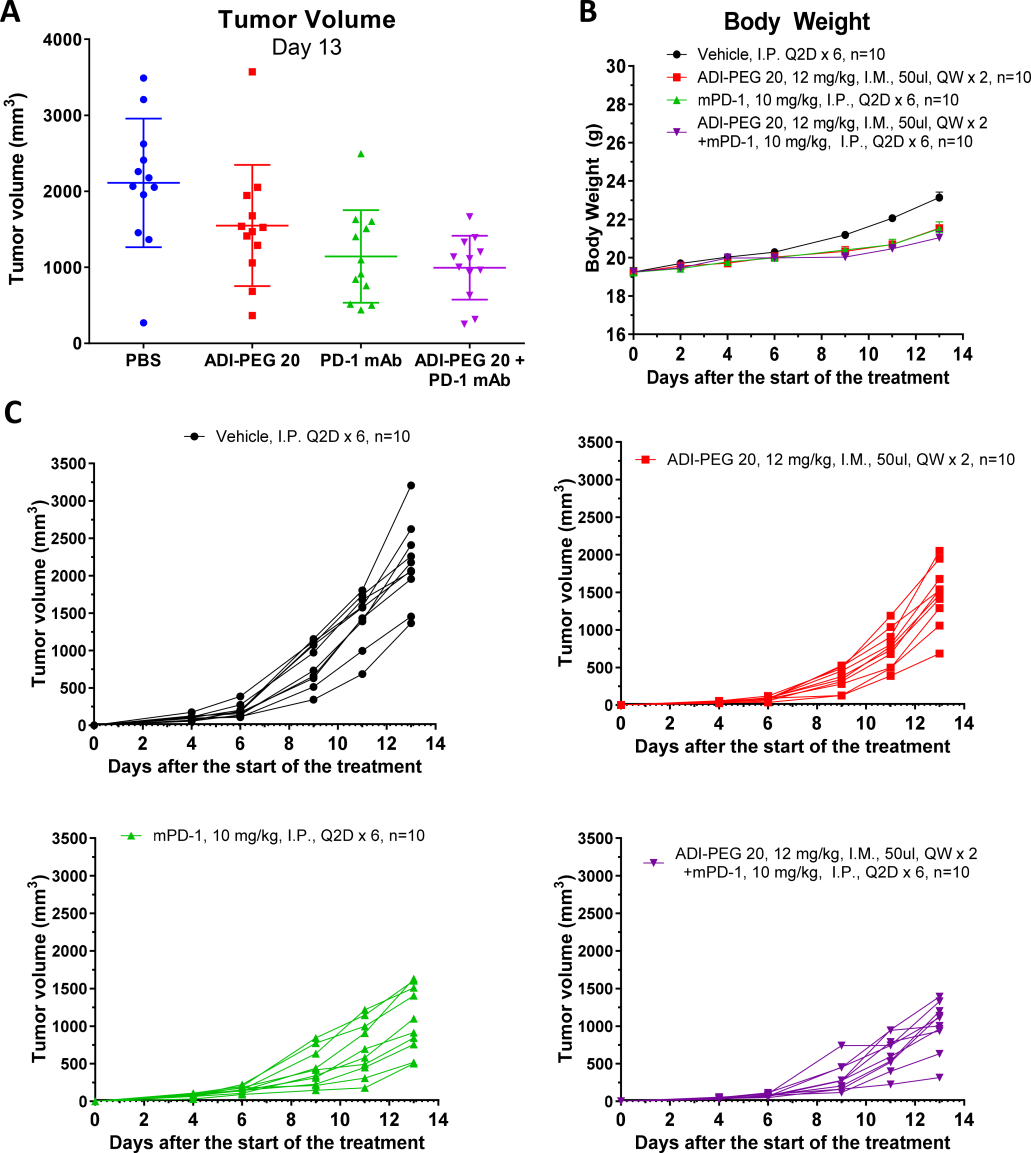
ADI-PEG 20 reduces B16-F10 tumor growth as a single agent and in combination with anti-mPD-1 mAb B16-F10 cells were implanted into C57BL/6J mice and the next day the mice were treated with 12 mg/kg of ADI-PEG 20 QWx2 IM, 10 mg/kg anti-mPD-1 Q2D IP, 12 mg/kg of ADI-PEG 20 QWx2 IM and 10 mg/kg anti-mPD-1 Q2D IP or vehicle (PBS) control. Tumor volume (**A** and **C**) and body weight (**B**) were monitored for two weeks.

**Figure 10:**
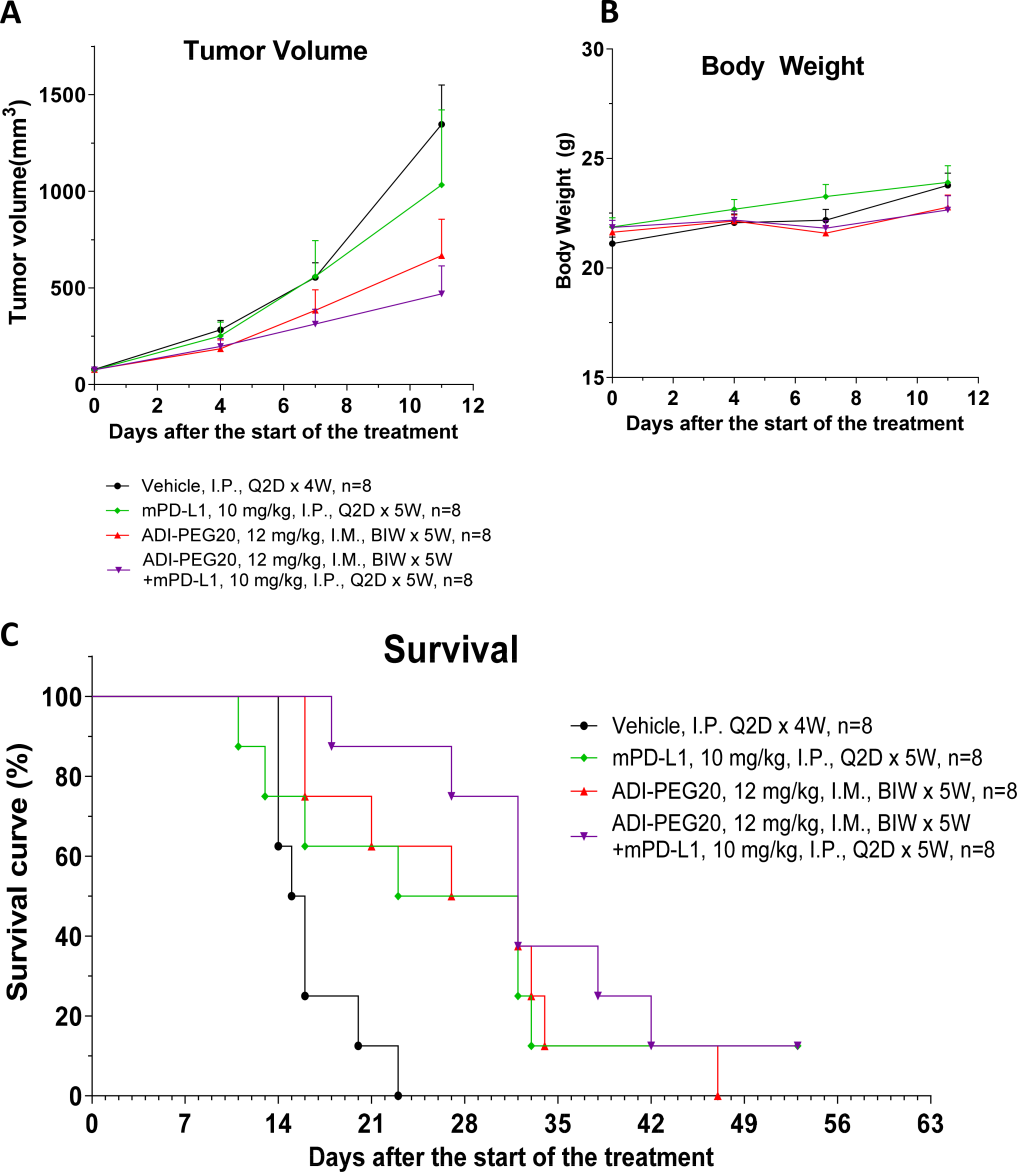
ADI-PEG 20 reduces CT26 tumor growth and prolongs survival as a single agent and in combination with anti-mPD-L1 mAb CT26 cells were implanted into Balb/c mice and the next day the mice were treated with 12 mg/kg of ADI-PEG 20 BIWx5W IM, 10 mg/kg anti-mPD-L1 Q2D x 5W IP, 12 mg/kg of ADI-PEG 20 IM BIWx5W and 10 mg/kg anti-mPD-L1 IP Q2D x 5W or vehicle (PBS) control. Tumor Volume (**A**), body weight (**B**) and survival (**C**) were assessed over time.

Next, we investigated whether ADI-PEG 20 can be effective in reducing tumor growth *in vivo* in immunocompetent models. In addition to studying effect of ADI-PEG 20 as a single agent we assessed if it would be beneficial to combine it with PD-1/PD-L1 neutralizing antibodies. B16-F10 melanoma and CT26 colon carcinoma models were used in these studies (conducted at a CRO). Both B16-F10 and CT26 are arginine auxotrophic cancers as ADI-PEG 20 inhibits their growth *in vitro* (Supplementary Figure 4A and 4B). *In vivo* studies were performed as described in Materials and Methods and results are shown in Figures 9 and 10.

In both B16-F10 and CT26 models ADI-PEG 20 slowed tumor growth and was well-tolerated. The B16-F10 model, as set up in the CRO where we have conducted the studies, is somewhat immunogenic and therefore anti-mouse PD-1 mAb was moderately effective in reducing tumor growth (*p* < 0.01). ADI-PEG 20 was less effective than anti-PD-1 mAb (not statistically significant) and only slightly reduced tumor growth (Figure 9). The combination of ADI-PEG 20 and anti-PD-1 mAb was more effective than either agent alone (not statistically significant).

In CT26 model ADI-PEG 20 had greater effect on tumor growth inhibition compared to anti-mPD-L1 antibody and their combination was better than either agent alone (not statistically significant). Survival analysis (mice were euthanized once their tumors reached ~ 3000 mm^3^) showed similar trends as more mice survived when treated with the combination of ADI-PEG 20 and anti-PD-L1 mAb (Figure 10C).

## DISCUSSION

ADI-PEG 20 can inhibit cell proliferation *in vitro* and tumor growth *in vivo* and is being investigated in clinical trials for ASS1-deficient tumors. To date ADI-PEG 20 has been demonstrated to be well-tolerated and shown promise in clinical studies [1–7].

To better understand potential impact of ADI-PEG 20 on immune microenvironment we have studied its effect on expression of immunosuppressive PD-L1 on tumor cells. PD-L1 levels can be affected by various stimuli, for instance, signaling through the MEK/ERK and the PI3K/AKT pathways has been shown to increase PD-L1 expression [30, 31] and Tsai et al. [32] have reported RAS/PI3K/ERK pathway activation by ADI-PEG 20 in some cancer cell lines.

When studying effects of ADI-PEG 20 on PD-L1 levels in a panel of cancer cell lines we compared its effects to those of INFg. Immune attack via IFNγ release leads to upregulation of PD-L1 creating an “immune shield” to protect normal mucosa from autoimmune attack in the setting of chronic inflammation or infection. Tumor cells have co-opted the PD-1/PD-L1 regulatory mechanism to avoid immunologic surveillance thereby facilitating cancer growth. INFg has been shown to induce PD-L1 in a number of cancer cells lines [33–36]. ADI-PEG 20 elicited a concentration-dependent increase in PD-L1 mRNA expression (Figure 2) which translated into increased PD-L1 surface protein expression (Figure 1). In the majority of the tested cell lines the magnitude of PD-L1 upregulation induced by ADI-PEG 20 was much smaller than that induced by IFNγ. The mechanism of PD-L1 upregulation that we have observed in certain cancer cell lines is not known. ADI-PEG 20 induced slight upregulation of PD-L1 it A375 cells where according to Tsai et al. [32] ADI-PEG 20 cannot activate RAS/PE3K/ERK pathway.

To learn how ADI-PEG 20 may affect T cells we treated healthy human PBMCs under resting conditions or when stimulated with anti-CD3/CD28 beads. PBMCs maintained high viability after ADI-PEG 20 treatment (Supplementary Figure 2), which is consistent with previous findings that arginine starvation does not affect T cell viability [17, 37].

When used at high concentrations that quickly convert all available media arginine into citrulline ADI-PEG 20 inhibited T cell activation progression past the initial stage, PBMC proliferation. Early activation marker CD69+ was upregulated on CD4+ and CD8+ T cells in the presence of ADI-PEG 20 and remained at high levels throughout our analyses (up to 72 h post stimulation). On the other hand CD25+ T cells and IL-2 and IFNγ secretion into the media were decreased by 5 nM ADI-PEG 20 when added at the beginning of PBMC stimulation. These results are consistent with previously reported findings by Munder et al. [37] who observed increased CD69 and decreased CD25 expression on T cells stimulated in arginine-free versus arginine-containing media. When 5 nM ADI-PEG 20 was added 24 h after stimulation was initiated it did not reduce IL-2 secretion and only mildly inhibited CD25 upregulation; delaying ADI-PEG 20 treatment by 48 h after stimulation or using it at lower concentrations (less than 1 nM) eliminated its effects on CD25 upregulation and cytokine secretion. Thus, complete absence of arginine during T cell stimulation can suppress their activation while presence of low arginine or its removal later in the process is not inhibitory.

While T cells can utilize citrulline to synthetize their own arginine this regeneration pathway may not be sufficient to satisfy high metabolic needs for arginine during priming. Geiger and colleagues recently showed that there is an abrupt decrease in intracellular arginine between 24 and 48 hours after T cell activation [38].

Upregulation of T cell exhaustion markers CTLA-4, PD-1 and PD-L1 was also inhibited by ADI-PEG 20 when present during PBMC stimulation.

CD4+ T cell differentiation into cells characterized by markers found on regulatory T cells (CD3+CD4+CD25+FoxP3+CTLA-4+) was particularly sensitive to ADI-PEG 20 treatment as even less than 1 nM concentrations suppressed accumulation of these cells (Figure 7). It appears that various T cell subsets have differential sensitivity to ADI-PEG 20 when it is present at the start of stimulation. As mentioned earlier ADI-PEG 20 does not appear to affect T cells when added before or after initial stimulation.

Thus, while complete depletion of arginine in lymph nodes during T cell priming would have negative effect on their activation low arginine concentrations are likely to be tolerated (especially in the presence of high citrulline) and may inhibit regulatory T cell accumulation. In tissues ADI-PEG 20 is unlikely to impact already activated T cells.

Further studies are needed to better understand consequences of ADI-PEG 20 treatment on tumor microenvironment *in vivo*. Apart from potential effects on T cells ADI-PEG 20 may affect myeloid cells as it competes for substrate with L-arginase and iNOS enzymes present in MDSCs and macrophages. L-arginine and L-citrulline have been shown to have differential effects on macrophages. When arginine is depleted in the media M1 macrophages produce NO from citrulline (transported from the media) using the arginine regeneration pathway. NO production from citrulline was not blocked by arginase as it was when L-arginine was present in the media [39].

To analyze how ADI-PEG 20 affects tumors in immunocompetent environment we studied its effect in syngeneic models. We conducted initial studies in-house using poorly immunogenic B16-F10 mouse melanoma model. B16-F10 cells have high ASS1 levels (Supplementary Figure 4C) however, ADI-PEG 20 inhibited their growth *in vitro* possibly due to a mutation in ASS1 or deficiency/mutation in another enzyme (e.g. argininosuccinate lyase) involved in the conversion of citrulline to arginine. Alternatively, B16-F10 cells may not be able to produce arginine from citrulline fast enough or deplete the nitrogen source needed to convert citrulline back to arginine.

IHC analysis of the B16-F10 tumor sections revealed that treatment with ADI-PEG 20 led to a marked increase in tumor infiltrating T cells. In contrast to the low level of tumor T cell infiltrate found in the non-treated mice, five out of six ADI-PEG 20 treated animals had a large number of T cells in their tumors (Figure 8), both CD4+ and CD8+ (data not shown). This suggests that ADI-PEG 20 may improve tumor immunogenicity. However, we don't know what caused this infiltration (possibly instigated by inflammation) or its exact composition and functional state.

We have conducted tumor growth inhibition studies at a CRO where B16-F10 model is moderately immunogenic and responds to the treatment with anti-mPD-1 mAb. ADI-PEG 20 was somewhat less efficacious than anti-PD-1 mAb in this model (not statistically significant). Importantly, combination of ADI-PEG 20 and anti-mPD-1 mAb appeared to be additive (Figure 9).

ADI-PEG 20 also reduced tumor growth and prolonged survival in another moderately immunogenic model, colon carcinoma CT26. This is also arginine auxotrophic cancer whose growth is inhibited by ADI-PEG 20 *in vitro* due to very low ASS1 levels (Supplementary Figure 3B and 3C). Combining ADI-PEG 20 with anti-mPD-L1 mAb *in vivo* resulted in superior tumor growth control and survival compared with either agent alone (not statistically significant) as shown in Figure 10. It is encouraging that ADI-PEG 20 has activity in immunocompetent models and seems to be additive with immune checkpoint blockers anti-PD-1 and anti-PD-L1 mAbs.

## MATERIALS AND METHODS

### Reagents

ADI-PEG 20 is a recombinant protein cloned from *Mycoplasma hominis* and subsequently produced in *Escherichia coli*. The recombinant protein is PEGylated with 20,000 MW PEG [40–42]. C397A substitution was used to generate the inactive ADI-PEG 20 mutant.

Frozen healthy donor PBMCs were purchased from AllCells. Cancer cell lines AGS, A2780, A375, HCT116, HT29, HT-1080, K562, SNU-1, SN-16, SNU-5, SNU-398, N87, A549, MSTO, SKOV-3, Mia-Paca-2, Panc-1, H1299 were purchased from ATCC.

TaqMan^®^ Gene Expression Cells-to-CT™ Kit, IFNγ, Live/Dead fixable stains (Green and Near-IR), PHA, AIM-V medium, Glutamax, CD3/CD28 Dynabeads, DynaMag-5, SimplyBlue^TM^ SafeStain, BCA assay kit, mouse anti-GAPDH monoclonal antibody HRP conjugate and SuperSignal West Femto maximum sensitivity substrate were obtained from ThermoFisher Scientific.

The following flow cytometry reagents were acquired from BD Bioscience - SB(BSA) buffer, human Fc block, human FoxP3 buffer set, AbC total antibody compensation bead kit, ArC amine reactive bead kit and antibodies specific to CD274-APC clone MIH1, CD279-PE clone MIH4, CD152-PE clone BNI3, CD3-PE-Cy7 clone SK7, CD8-FITC clone SK1, CD4-PerCP-Cy5.5 clone SK3, CD69-APC clone FN50, CD25-APC clone M-A251, CD127-PE clone HIL-7-M21, FoxP3-Alexa-488. CD274-PE clone 29E.2A3 and CFSE were purchased from BioLegend.

RIPA buffer and protease inhibitors were purchased from Sigma.

Goat anti-mouse IgG-HRP was from Santa Cruz Biotech.

For IHC studies the following antibodies were used: anti-CD3 rabbit mAb [SP7] from Abcam, anti-CD4 polyclonal Guinea pig antiserum and anti-CD8a polyclonal rabbit antibodies were from SYSY, whole IgG donkey anti-rabbit and anti-guinea pig were from Jackson ImmunoResearch.

### PMBC treatment

PBMCs from five different donors were tested in six repeat experiments. PBMCs were thawed, washed and rested overnight (18 h). The next day cells were collected, resuspended in AIM-V medium supplemented with Glutamax and counted. Cell concentration was adjusted to 2 × 10^6 cells per mL and 2 mL of cells were added per well of a 6-well plate. PBMCs were treated with 0, 0.8, 4 or 20 nM of ADI-PEG 20 in the presence or absence of CD3/CD28 Dynabeads or PHA. CD3/CD28 Dynabeads were prepared according to manufacturer's instructions. Cells were analyzed after 24 h, 48 h and 72 h of treatment.

Citrulline and arginine levels in the media were measured by LC/MS/MS.

IL-2 was measured by human IL-2 ELISA kit and IFNγ was measured by human IFNγ ELISA MAX Deluxe kit (BioLegend).

### Cancer cell lines treatment

Cancer cell lines were cultured according to manufacturer's instructions. Cells were treated with increasing concentrations of ADI-PEG 20 and analyzed after 24 h, 48 h or 72 h incubation.

The effect of ADI-PEG 20 on cell viability and caspase 3/7 induction was analyzed by ApoTox-Glo triplex assay (Promega).

### Flow cytometry analysis

### PBMC

Prior to flow cytometry analysis Dynabeads were removed from samples. Cells were blocked in human Fc block, stained with Live/Dead fixable Near-IR stain followed by appropriate antibody solution mixes. For effector T cell (Teff) analyses antibody mix contained: CD3-PE-Cy7, CD8-FITC, CD4-PerCP-Cy5.5, CD69-APC and either CD274-PE, CD279-PE or CD152-PE. For analysis of regulatory T cells (Treg) antibody mix included CD3-PE-Cy7, CD4-PerCP-Cy5.5, CD25-APC and either CD127-PE, CD274-PE, CD279-PE or CD152-PE. After surface staining samples were washed, those stained with Teff antibody mix were immediately analyzed by flow cytometry, while samples stained with Treg antibody mix were permeabilized with human FoxP3 buffer set and intracellularly stained with FoxP3-FITC antibody. Fifty thousand events were acquired for each sample on a Guava EasyCyte 8HT (Millipore). Data acquisition and analysis were performed with InCyte software (Millipore). AbC total antibody compensation bead kit and ArC amine reactive bead kit were used to create single stained fluorophore controls and perform spectral overlap compensation on the cytometer.

Gating strategy: Singlet gate was determined by FSC-H versus FSC-A plot. Fluorescent minus one (FMO) controls were used to set up other analyses gates. Singlet cells were gated on live cells based on Live/Dead fixable Near-IR stain and singlet and live cells were further analyzed for the presence of CD3 T cell marker. Within CD3+ live and singlet cells CD4+ and CD8+ gates were set up, then each of these populations were analyzed for the presence of other markers - CD69, CD274, CD279, CD152. For Treg analysis singlet and live CD3+CD4+ cells were analyzed for the presence of CD25, FoxP3, CD127, CD274, CD279 and CD152 markers.

For proliferation studies PMBCs were labeled with CFSE prior to the treatment. Briefly, CFSE was diluted to 5 μM in PBS, cells were resuspended in CFSE solution and incubated for 20 minutes at 37°C. The dye was quenched with 5 volumes of culture media with 10% FBS. Cells were pelleted and resuspended in pre-warmed culture medium.

### PD-L1 surface levels in cancer cell lines

Cell were collected and washed in cold SB(BSA) buffer. Then cells were resuspended in 80 μL SB(BSA) buffer and incubated at room temperature for at least 10 min. Each sample was split in two (40 μL each) for specific staining with anti-PD-L1 mAb and to assess non-specific binding with isotype control antibody. Live/Dead fixable green stain was prepared according to manufacturer's instructions and the solubilized stain was diluted 1:10 in SB(BSA) buffer. Then 0.5 μl of the diluted Live/Dead fixable green stain and either 2.5 μL anti-CD274-APC mAb + 7.5 μL SB(BSA) or 10 μL isotype control-APC mAb was added to each sample. Samples were incubated on ice protected from light for 35 min, washed twice in SB(BSA) buffer and resuspended in 150 μL of SB(BSA) buffer. Non-stained and single stained controls were used to adjust instrument settings. Ten thousand events were acquired for each sample on a Guava EasyCyte 8HT (Millipore). Data acquisition and analysis were performed with InCyte software (Millipore). Cells were gated on singlets (determined by FSC-H versus FSC-A) and live cells within the singlet gate were analyzed for PD-L1 levels. Percent PD-L1 positive and mean and median fluorescent intensity were determined by subtracting background signal determined with isotype control antibody from specific signal obtained with anti-PD-L1 antibody.

### RT-qPCR

RT-qPCR was conducted with TaqMan^®^ Gene Expression Cells-to-CT^™^ Kit according manufacturer instructions. The qPCR step was performed with primer-probe mixes purchased from IDT multiplexing an assay for gene of interest (CD274 or ASS1) with housekeeping beta-actin assay (for normalization). The CD274 probe was labeled with FAM (assay name Hs. PT.58.4665575), the ASS1 probe was labeled with Hex (assay name Hs. PT.56a.2920438) and the beta-actin probe was labeled with Cy5 (assay name Hs. PT.39a.22214847). Multiplexing did not affect assay performance - each of the above assays performed similarly when run as a singleplex or as a multiplex of beta-actin with either CD274 or ASS1. The qPCR was conducted in an ABI 7500 real time PCR system with the following program: 1 cycle at 50°C for 2 min, 1 cycle at 95°C for 10 min and 40 cycles of 15 sec at 95°C followed by 1 min at 60°C.

Control reactions without reverse transcriptase or a template were included to verify absence of genomic DNA or template contamination. Samples were run in duplicate during the qPCR step.

### Western blotting

Cells were lysed in RIPA buffer supplemented with protease inhibitors. Lysate protein concentrations were determined by BCA assay and ~20 μg of protein was loaded for each sample onto 4–12% Tris-glycine polyacrylamide gels, two identical gels were used – one was stained with Simply Blue to confirm the protein amount loaded and another was transferred onto PVDF membrane with iBlot Western Blotting System (Thermo-Fisher Scientific). The membranes were blocked with 5% non-dry milk-TBST and probed with primary antibodies (anti-ASS1 mAb or GAPDH mAb) followed by incubation with secondary goat anti-mouse IgG antibody conjugated with HRP. Detection was conducted with SuperSignal West Femto maximum sensitivity substrate and both the blots and stained protein gels were imaged on a BioRad's ChemiDoc Imager and band intensities were quantified using Image Lab software.

### *In vivo* studies

5 × 10^5 B16-F10 cells were implanted subcutaneously into the right flank of C57BL/6 mice in 0.1 mL PBS. Animals were randomized into two groups – four mice into the control group and six mice into the ADI-PEG 20 treatment group. The day after tumor inoculation and again 6 days later (7 days post tumor implantation) the control animals were injected intramuscularly with vehicle (PBS) and mice in the experimental group were injected intramuscularly with 12 mg/kg of ADI-PEG 20, both in a fixed 40 μL total volume. Animals were euthanized on Day 7 after the last treatment (14 days post tumor implantation) and their tumors were excised. Tumors tissues were preserved in 4% paraformaldehyde solution for at least 24 hours and then embedded into paraffin.

IHC staining was performed at Zyagen: after deparaffinization, sections were boiled in 10 mM citrate buffer under pressure for 5 minutes and after cooling down, sections were blocked with 5% BSA in PBS for 2 hours. Sections were then incubated with anti-CD3 mAb diluted 1:100 in 1% BSA in PBS at 4°C in a humidified chamber. Secondary antibody, anti-rabbit donkey sera conjugated with Alexa-Fluor-488 was diluted 1:100. Sections were counterstained with DAPI for 2 minutes.

Tumor growth and survival studies were performed at Wuxi AppTec. Anti-mouse PD-1 and PD-L1 monoclonal antibodies were purchased from BioXcell.

C57BL/6J mice were inoculated subcutaneously at the right flank with 2 × 10^5 B16F10 tumor cells in 0.1 mL of PBS (containing 0.05 mL matrigel) The animals were randomized based on the body weight, and treatment was started the next day after the cell inoculation with 12 mg/kg of ADI-PEG 20 QWx2 IM, 10 mg/kg anti-mPD-1 Q2D IP, 12 mg/kg of ADI-PEG 20 QWx2 IM and 10 mg/kg anti-mPD-1 Q2D IP or vehicle (PBS) control.

Balb/c mice were inoculated subcutaneously at the right flank with 2 × 10^5 CT26 tumor cells) in 0.1 mL of PBS. Ten days after the tumor implantation the mice were randomized into four groups based on the tumor size and treated with 12 mg/kg of ADI-PEG 20 BIWx5W IM, 10 mg/kg anti-mPD-L1 Q2D x 5W IP, 12 mg/kg of ADI-PEG 20 IM BIWx5W and 10 mg/kg anti-mPD-L1 IP Q2D x 5W or vehicle (PBS) control.

Tumor size was measured in two dimensions using a caliper, and the volume was expressed in mm^3^ using the formula: V = 0.5 a × b^2^ where a and b are the long and short diameters of the tumor, respectively.

### Statistical analysis

One-way ANOVA was performed to compare different treatments using GraphPad.

## SUPPLEMENTARY MATERIALS FIGURES


