# Supplementary material for: PEGylated arginine deiminase can modulate tumor immune microenvironment by affecting immune checkpoint expression, decreasing regulatory T cell accumulation and inducing tumor T cell infiltration
Source: Oncotarget. 2017 Jul 26;8(35):58948–63. doi: 10.18632/oncotarget.19564 (PMC5601705; doi:10.18632/oncotarget.19564)
Supplement: Supplementary file 1 [file oncotarget-08-58948-s001.pdf]

PEGylated arginine deiminase can modulate tumor immune microenvironment by affecting immune checkpoint expression, decreasing regulatory T cell accumulation and inducing tumor T cell infiltration

SUPPLEMENTARY MATERIALS

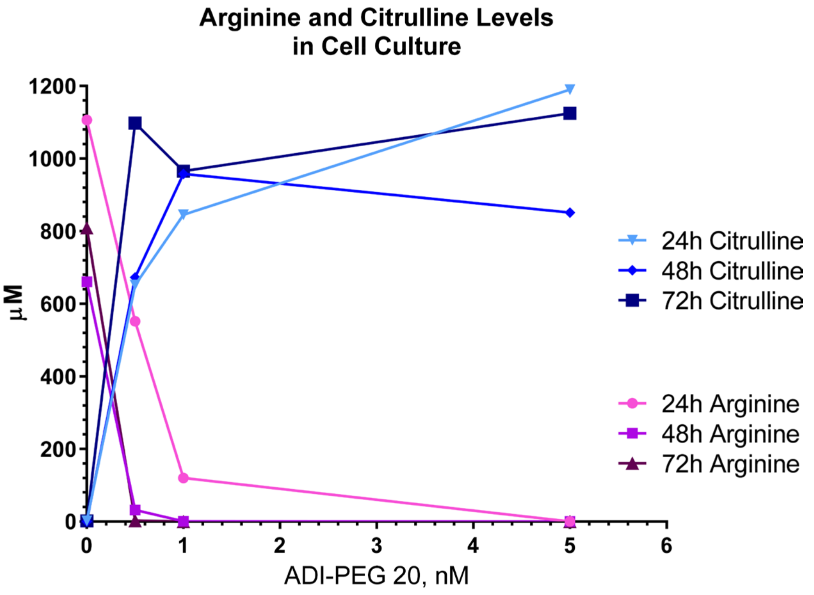

Supplementary Figure 1: Arginine and citrulline levels in the media overtime in the presence of 0-5 nM ADI-PEG 20.

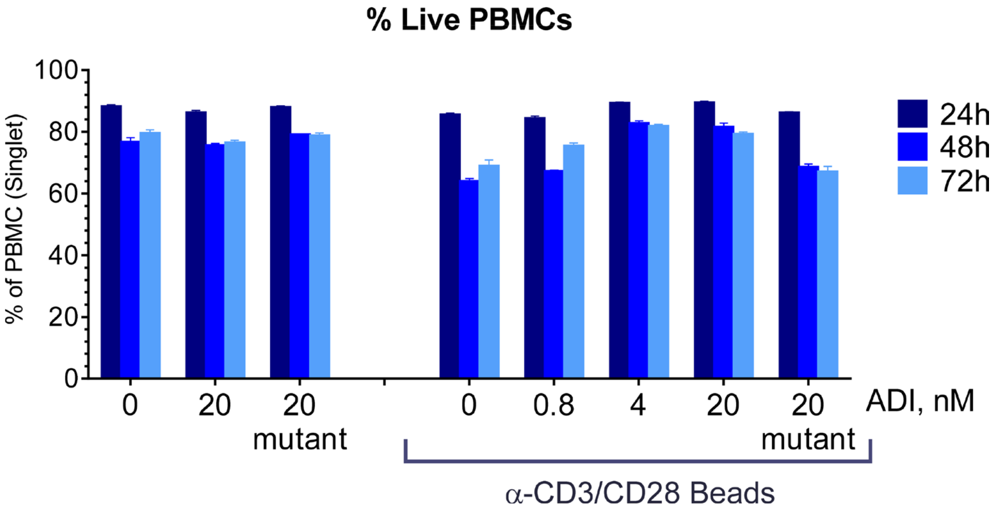

Supplementary Figure 2: PBMCs remain viable in the presence of ADI-PEG 20. PBMCs were stimulated with anti-CD3/CD28 Dynabeads in the presence or absence of ADI-PEG 20 or mutant ADI-PEG 20. Percentages of viable cells were determined by flow cytometry at 24 h, 48 h & 72 h.

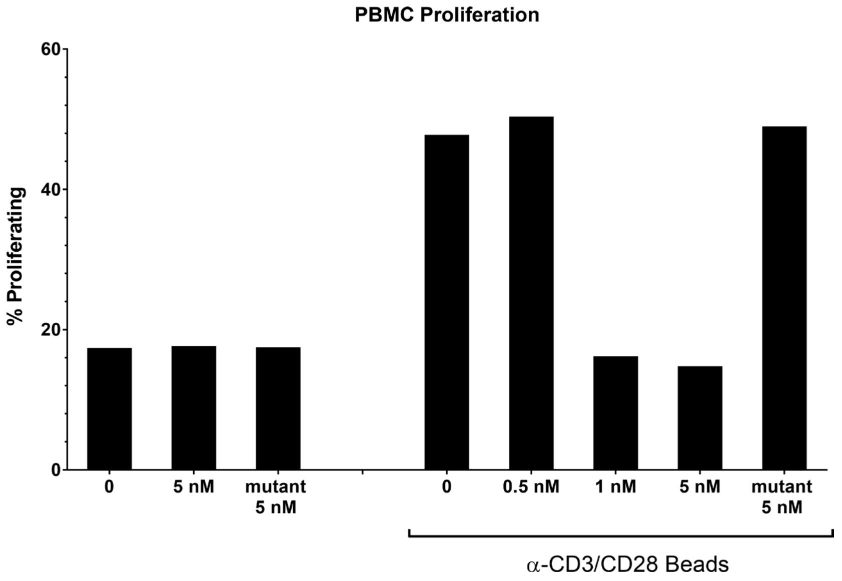

Supplementary Figure 3: PBMCs proliferation in the presence of ADI-PEG 20. PBMCs were stained with CFSE and stimulated with anti-CD3/CD28 Dynabeads in the presence or absence of ADI-PEG 20 or mutant ADI-PEG 20. Percentages of proliferating cells were determined by flow cytometry after 72 h incubation.

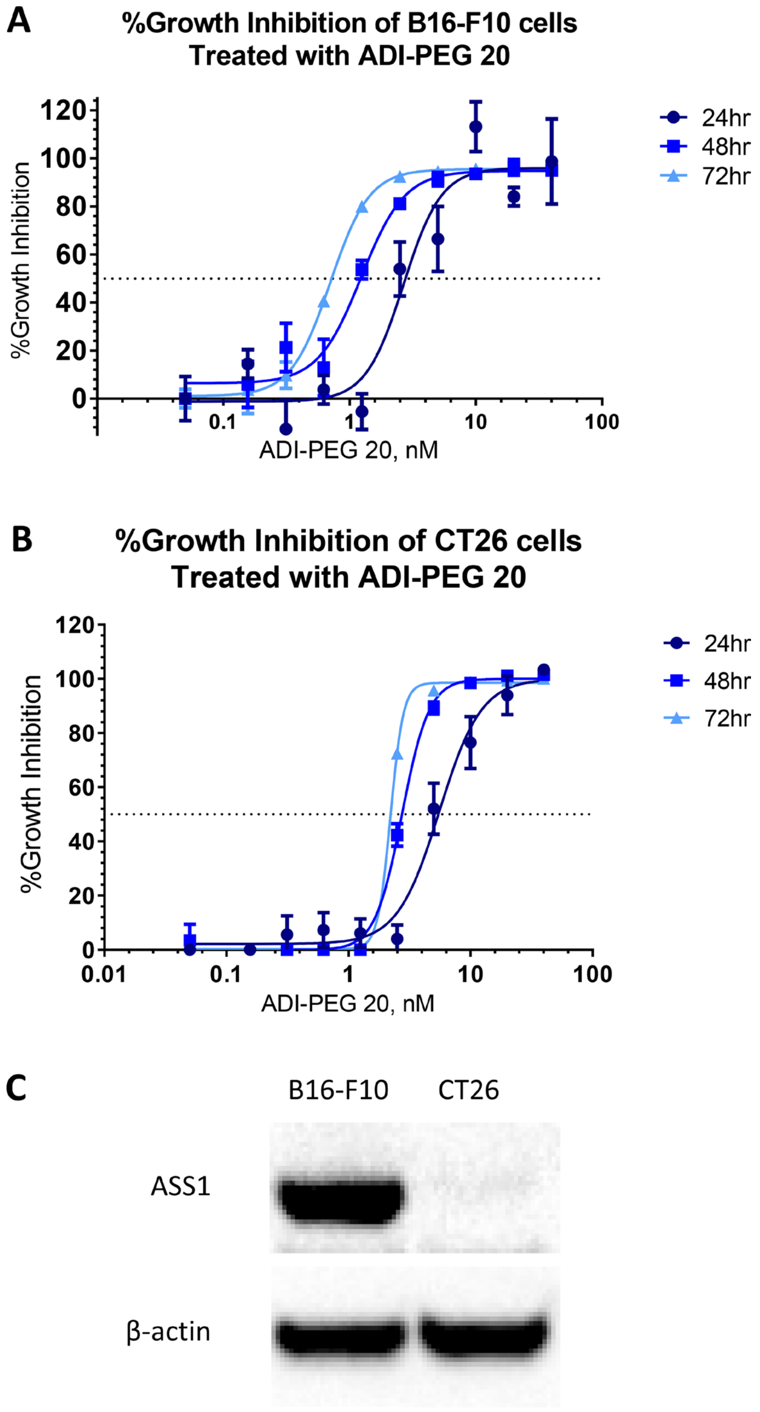

Supplementary Figure 4: ADI-PEG 20 inhibits growth of B16-F10 (**A**) and CT26 (**B**) cells *in vitro*. B16-F10 and CT26 cells were treated with ADI-PEG 20 and their viability was assessed at 24 h, 48 h & 72 h using Cell-Titer Fluro (Promega) and compared to the viability at the beginning of the treatment to determine % growth inhibition. % Growth Inhibition = 1 – ((Sample Viability – Viability @ T0)/(Non-treated Control Viability – Viability @T0)). Viability @T0 is cell viability at the start of the treatment. ASS1 levels in B16-F10 and CT26 cells were assessed by Western Blotting (**C**).
